# “Fear can hold you, hope can set you free”. Analysis of Italian prisoner narrative experience of the COVID-19 pandemic

**DOI:** 10.1108/IJPH-07-2020-0051

**Published:** 2021-08-14

**Authors:** Antonia Sorge, Federica Bassanini, Jennifer Zucca, Emanuela Saita

**Affiliations:** Department of Psychology, Catholic University of the Sacred Heart, Milan, Italy

**Keywords:** Italy, Prison, Qualitative research, Psychological distress, Covid-19, Pandemic

## Abstract

**Purpose:**

This study aims to explore the psychological effects of lockdown during the early stages of the COVID-19 pandemic on people living in an Italian prison. The suspension of family visits and most activities, along with the difficulties in applying social distancing to this vulnerable population was associated with increased psychological distress. Riots broke out over two days in more than 22 prisons across Italy at the beginning of March 2020, highlighting the negative psychological impact of the pandemic and the country’s emergency policies.

**Design/methodology/approach:**

The research involves 17 men (Italians and foreigners) detained in a Lombardy prison from 1 March to 4 May 2020, corresponding to the lockdown phase in Italy. The qualitative content analysis (CA) of 27 posts, written by participants during that period and published on the blog “L’Oblò”, were analysed. The analysis allowed the identification of topics and subtopics that are related to two major categories of content: cognitions and emotional connotations about the COVID-19 lockdown in prison.

**Findings:**

Analysis showed that blog post content was predominately negative in terms of emotional connotations. The most frequent coded negative emotional connotations were: missing, worry, psychological pain and fear, whilst the most frequent coded positive emotional connotations were: hope and gratitude for the support they received from prison workers. The rest of the blog content was coded as “cognitions”. Cognitions were coded as descriptions of lockdown effect on detention; prison during the COVID-19 emergency; the pandemic situation in general; and comparison between inside and outside prison.

**Originality/value:**

The current study is original as it describes through blog CA the psychological condition of prisoners during the first COVID-19 pandemic lockdown in the most affected region in Italy.

## Introduction

Since the end of February 2020, Italy (especially the Lombardy region) has witnessed the rapid spread of COVID-19. Afterwards, on 11 March, the World Health Organization (WHO) declared it a pandemic. On 8 March 2020, the Civil Protection Department in Lombardy declared the presence of 3,372 positive cases of COVID-19, an increase of 630 compared to the previous day. A total of 267 people had died (a further increase of 113) with 4,189 total cases (an increase of 769) (Protezione Civile, 2020). On the same day, total cases in Italy were 7,243, which at the time accounted for almost 60% of the total cases in Europe (12,226) and 23% of the cases worldwide (31,303) (ECDC, 2020).

Following the rapid increase of COVID-19 infections, the Italian Government promptly intervened with preventive and containment measures throughout the country, including prisons. The preventive and containment measures applied in prison (described below) were taken before the publication of Europe’s Regional WHO Office’s guidance: “preparedness, prevention and control of COVID-19 in prisons and other places of detention”, published on 15 March 2020 (WHO, 2020).

The urgency in adopting preventive and restrictive measures was related to the closed nature of the prison setting which increases the risk of infections. Pathogens like COVID-19, transmitted from person to person via droplets or close contact, are extremely likely to spread rapidly in prison – due to environmental factors such as overcrowding, poor ventilation, close habitation or dormitory-style housing and poor hygienic conditions (Montoya-Barthelemy *et al.*, 2020; Dutheil *et al.*, 2020; Dolan *et al.*, 2016). Furthermore, the underlying health conditions of people in prison (e.g. chronic diseases and mental health illnesses) make them more susceptible to contracting severe infectious diseases (Wurcel *et al.*, 2020; Crowley *et al.*, 2018; Fazel and Baillargeon, 2011).

For these reasons, anti-contamination measures must be strongly adhered to in prisons (Kinner *et al.*, 2020). On 26 February 2020, the Head of the Italian Department of Prison Administration launched widespread awareness and information-raising activities concerning the virus. In addition to the instructions already provided by the Ministry of Health (i.e. use of protective equipment, disinfection procedures and physical distancing) the Department of Prison Administration chose to adopt the following restrictive measures (Ministero della Giustizia, 2020):

- suspension of release on temporary licence and semi-freedom regime;
- suspension of group treatments;
- suspension of activities for which external community access is foreseen or necessary;
- containment of external and internal work activities for which the presence of people from outside is foreseen; and
- replacement of visits with family members or third parties with remote correspondence calls using the equipment provided in prison (Skype) and telephone.

Time allowed outside the cells was also significantly reduced to limit contact between people in prison as much as possible.

On 7 March 2020, the Lombardy region declared lockdown (i.e. an emergency protocol that included the order to stay at home, closure of public spaces, schools and most workplaces). It was followed by a nationwide lockdown two days later, indicating a worsening situation. People in Italy could only move around for grocery shopping, doctor’s visits or going to work. A self-certification form was required by all detailing the reason for movement and travelling within or outside of the city was prohibited.

Following the declaration of the lockdown, the Italian penitentiary system was heavily shaken by serious riots (Buffa, 2020; Cingolani *et al.*, 2020). The violent riots originally broke out in Lombardy and spread to 22 prisons all over Italy including Foggia, Modena, Rome Rebibbia, Rome Regina Coeli, Bologna, Melfi, Rieti, Bari, Palermo, Santa Maria Capua Vetere, Velletri, Prato, Milan San Vittore, Matera, Chieti, Ivrea, Caltanissetta, Enna, La Spezia, Ferrara, Termini Imerese, Trani and Potenza between 7 and 9 March 2020. These riots involved roughly 6,000 prisoners and caused considerable damage to prison facilities and injuring dozens of people including 40 prison officers. It was observed that 12 prisoners died from overdoses of psychotropic drugs and methadone stolen from prison pharmacies during the riots [1] [2]. The riots were in response to the implementation of restrictive measures, which included the interruption of external visits, uncertainty and fear of the situation.

San Vittore, which is in the heart of Milan, is the largest correctional facility in Lombardy. It was the site of one of the most violent riots that broke out at the beginning of March 2020. It hosts mainly people on pre-trial detention and has a capacity of 799 with a section for men and another for women prisoners. The prison has a star-shaped structure with six wings that correspond to the six sections of the prison. At the time of the COVID-19 outbreak in Lombardy, San Vittore housed about 1,029 people in prison (931 men and 98 women) with an overcrowding rate of 129% (Ministero della Giustizia, 2020b).

San Vittore represented an important site during the lockdown. Following the first positive cases in prison at the end of March, “DOCTORS WITHOUT BORDERS” in collaboration with the management of San Vittore, set up a centre for the treatment of COVID-19 within the prison, becoming the reference hub in Lombardy for the management of prisoners testing positive. COVID-19 positive people from other prisons were transferred to San Vittore to be placed under observation and monitored. The San Vittore initiative was later extended to other prisons in Lombardy, Marche, Piedmont and Liguria [3].

Currently, there is little research on the effect of the COVID-19 pandemic on prisoners (Carvalho *et al.*, 2020). To date, studies have focused mainly on the effects of isolation and quarantine (i.e. the separation and restriction of movement of people who have potentially been exposed to a contagious disease) among the general population (Brooks *et al.*, 2020; Loades *et al.*, 2020; Chtourou *et al.*, 2020; Clemente-Suárez *et al.*, 2020). A review on the psychological impact of quarantine among the general population highlighted that separation from loved ones, inadequate information and supplies, the loss of freedom, uncertainty over disease status, fears of infection and boredom are predictors of psychological distress and disorder (Brooks *et al.*, 2020).

The addition of such factors to those already deprived of their liberty (i.e. prisoners) further increases their psychological and behavioural reactions compared to people in the community (WHO, 2020). Suspension of visits (including volunteers, members of charities, trainees, students, etc.) reduces contact with the outside world and might produce the use of maladaptive coping strategies due to the perception of poor social support (Hewson *et al.*, 2020b).

During the months of lockdown, people in the community have engaged in creative activities to avoid succumbing to boredom and lack of contact with other people. Much use has been made of the internet, smartphones and social networks. However, substitute activities are scarce in prisons, where access to the internet is restricted. The scarcity of activities, increased time spent in cells (about 23 h/day) and little or no contact with other people and the outside world, mimic conditions of solitary confinement. Even short periods in solitary confinement are associated with psychological consequences, including anger, depression, anxiety, paranoia, psychosis and exacerbation of underlying mental illness (Hewson *et al.*, 2020; Shalev and Edgar, 2015). Liebrenz *et al.* (2020) highlight that due to the high prevalence of mental and physical illness (Fazel and Seewald, 2012) and drug addiction among people in prisons (EMCDDA, 2019), putting them in quarantine and depriving them of visits from their psychiatrists and families can worsen the situation.

If quarantine or isolation is essential, then it is equally important to take every measure to ensure that this experience is as tolerable as possible for people (Brooks *et al.*, 2020). This can be achieved by providing knowledge about what is happening and why, providing meaningful activities, ensuring supplies such as masks, disinfectants and regular sanitizing materials (especially in a context of prison overcrowding) and continuing to provide psychiatric and psychological support. Recent studies underlined the difference in the development of psychological distress between quarantined people in prison and the general population (Kothari *et al.*, 2020; Okoro *et al.*, 2020). Okoro *et al.* (2020) observed that the high level of psychological distress among people in prison can stem from a poorer level of knowledge about COVID-19, sometimes associated with lower levels of education compared to the general population.

Starting from these considerations, the current study focuses on the psychological consequences of the COVID-19 lockdown among prisoners in a Lombardic prison, with an assumption that the situation may have contributed to greater psychological distress.

Given the impossibility of entering prison, the authors chose to focus on the qualitative content analysis (CA) of posts written by the people in San Vittore Prison (Milan, Italy) posted on their institutional blog “L’Oblò” (“The Porthole”) during the early stage of the COVID-19 pandemic in Italy. By analysing blog post content, it was possible to gain a thorough understanding of prisoner experiences without conducting interviews.

### Ethical consideration

The ethical handling of virtual data has been widely discussed (Markham *et al.*, 2012). In principle, blogs are in the public domain, but some consider this space private. Even though the Economic and Social Research Council (ESRC) Framework for Research Ethics (ESRC, 2015) considers information publicly shared on the internet as a public domain, the nature of information or communication through social networks requires a critical examination of privacy protection for those involved (SAGE, 2018). Therefore, despite the public nature of the blog, blog authors were contacted through email where the aim and conduct of the study were explained. The authors were also informed that participation was entirely optional. The service that runs the blog authorized the use of the material and informed consent was obtained from each writer. One participant did not consent to the sensitive data disclosure agreement (name, age and type of offence). Pseudonyms were used in this report to protect privacy. Data were collected between March and May 2020 through the examination of the blog “L’Oblò”, managed by professional staff in “La Nave” (“The Ship”), the department of San Vittore prison (Milan, Italy).

## Method

### Participants

The sample of our study included 17 male prisoners’ narratives published on a public blog. The mean age sample was 41.69 (SD = 12.00; range: 25–59). Most were Italian (*n* = 11), followed by Albanian (*n* = 4), one person was Kosovar and another was Egyptian. Reasons for conviction were reclassified as: crimes against persons (*n* = 6); drug-related crimes (*n* = 6); crimes against property (*n* = 4). In total, 3 out of 17 participants were in pre-trial detention, whilst the remaining 14 had yet to receive a final sentence. Among the 14 temporarily sentenced, the average prison sentence was 4.71 years (SD = 2.23; range = 2–8). All participants suffered from substance abuse disorders (cocaine = 10, heroin = 4, alcohol = 2).

### La Nave and its activities

All participants were detained in the ward called “La Nave” (“The Ship”) located on the fourth floor of the third wing of San Vittore prison. Although the riot in San Vittore prison broke out in the third wing, and therefore, also involved the “La Nave” ward, none of the participants in our study were involved in the riots. People in this ward can leave their cells from 8 a.m. till 8 p.m. The ward normally has a capacity of 60 people, but during the lockdown period in Italy, there were about 40 people (some were moved to other prisons whilst others were released to reduce the risk of contagion). In this ward, people participate in a nationwide substance dependence treatment programme run by a multidisciplinary team.

Socio-educational activities are carried out (e.g. motivation group), psychotherapeutic (e.g. psychological support group), cultural (e.g. the editorial staff of L’Oblò) and rehabilitative (e.g. choir performances twice a year during the festive periods). The programme is focused on those who are assessed as suitable and sufficiently motivated to undertake a path of change in a context of care and empowerment. People who participate in these programmes are asked to sign an agreement that requires them to respect the programme, the prison workers and prison rules. Those who are more able to deal with potentially stressful situations and who are involved in numerous individual and group activities are more likely to be allowed to participate. The programmes aim to encourage understanding and consider the behaviours that led both to addiction and deviance.

### The blog

“L’Oblò” [4] is one of the most important Italian blogs, managed and written by both the prisoners and prison workers of “La Nave” (“The ship”) ward in San Vittore prison (Milan, Italy). It was created in 2002, a few months after the ward opened. It is freely accessible and is updated monthly. Anyone hosted in the ward can contribute to the blog and they are free to write and share their reflections, thoughts, emotions and stories with the readers of “L’Oblò” without restrictions. They write on paper because they are not allowed to directly access the internet. Writings are then collected by the professionals of La Nave and published online. Prison workers write about events, activities, publish photos or communicate information. The blog’s content is also published as a free periodical with the financial contribution of “Feltrinelli Editore”. Although there are other Italian blogs published by people in prison (e.g. “Dentro e Fuori”), we chose to focus on the posts of “L’Oblò” because it is the only blog written by prisoners affected by riots during the March 2020 COVID-19 lockdown.

### Analysis strategy

Qualitative CA was performed on the blog posts published on “L’Oblò”. CA has been used in different fields of psychology to study topics such as emotional expression, casual explanations, stress and coping, anxiety, stigma, depression, cognitive and attributional lifestyles and moods. CA extracts desired information from a body of verbal material by systematically and objectively identifying categories and dimensions (Smith, 2000). An empirical and inductive approach to the coding system was used because of the exploratory and preliminary nature of this research as it allowed for the emergence of categories without preconceived influences. Within a qualitative methodology of research, CA of the narrative texts was used, meaning that the posts on the blog were considered narratives.

The objective of the coding scheme was to describe the topics addressed in the blog posts and identify categories of themes relating to the psychological consequences of further segregation experienced during the preliminary phase of the COVID-19 pandemic in Italy. We assumed that the restrictive measures (listed above) applied in prisons to contain the spread of the virus and the national lockdown had repercussions on the daily lives of prisoners from both a practical and psychological perspective.

Only posts published on the blog from 1 March to 4 May were included for CA. This specific period was chosen because it corresponds to the first lockdown phase that affected Italy during the COVID-19 pandemic. Despite the thematic frame, posts included different text types (i.e. information, documents, diaries, opinions and activity reports).

To be included in the CA blogs had to meet the following criteria:

- written by prisoners;
- related to COVID-19; and
- have a first-person experience during the lockdown as the main subject.

Posts selected were converted into a single body of text and read and analysed line-by-line by two authors independently (FB and AS). Major categories were assigned inductively when the topic and subtopics were identified and coded. Similar categories were grouped. For example, thoughts, ideas, opinions and explanations were grouped under the category “cognition”. Where interpretations of FB and AS differed, discussions with the other two authors (ES and JZ) occurred until consensus was reached. This process was iterative, with the authors frequently returning to the original documents to check that emerging topics and subtopics were consistent with the participant’s narratives.

**Figure 1:**
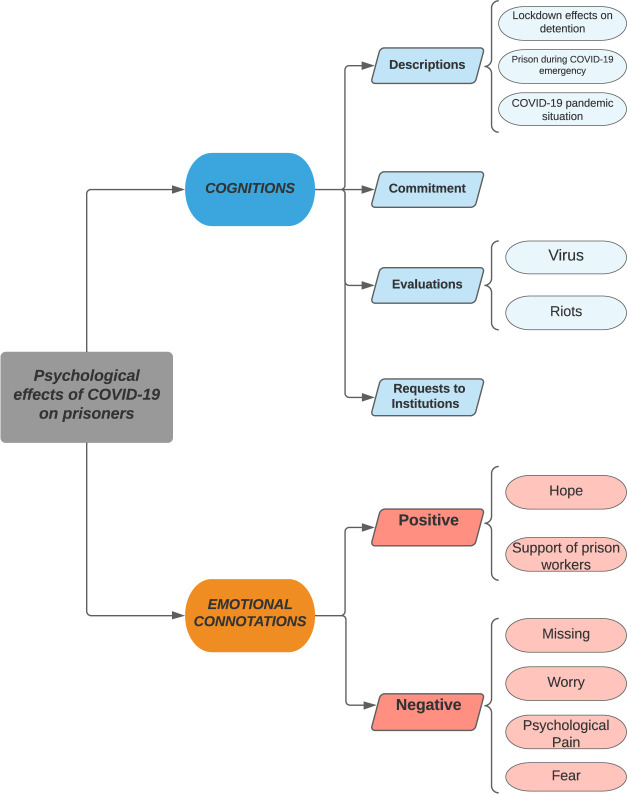
Flow-chart. Topics and subtopics related to the major categories

Frequency analysis for a blog topic and subtopic occurrences were calculated. Then, they were inductively assigned to one of two topic categories labelled “cognition” and “emotional connotations” concerning their content. For illustrative purposes, we have reported some examples of these in the results section for each topic and subtopic. Information was then synthesized into a flowchart (Figure 1). The flowchart includes topics and subtopics that occur more than five times. The topic categories are highlighted and linked to their subtopics – i.e. cognition: descriptions, commitment, evaluations and requests to institutions (e.g. the Italian Minister of Justice) and emotional connotations: positive and negative. Whilst the blogs were originally written in Italian, they were translated into English for this publication preserving the gist of the original posts.

## Results

According to the inclusion criteria, 27 out of 35 posts published on the blog during the established period were included. Posts were written by 17 separate individuals and varied in length from 98 to 484 words (M = 200, 4; SD = 101, 848). Nine posts were excluded due to the following reasons:

- articles did not relate to COVID-19;
- articles were written by professionals; and
- articles did not have the first-person experience of the prisoner during the lockdown as their main subject.

CA of the posts allowed us to describe the psychological conditions of people living in a Lombardy prison during the early phase of the COVID-19 pandemic. The content of the 27 posts included in this study has been divided into two major categories based on how people chose to write about their experience, i.e. from a more cognitive or emotional perspective.

The CA showed that “emotional connotations” (*n* = 94) were more common than “cognitions” (*n* = 79), and that emotional connotations were more likely to be negative (65/94) than positive (29/94). The most frequent negative emotional connotations were: missing, worry, psychological pain and fear, whilst the most frequent positive emotional connotations were hope and gratitude for the support received from prison workers. The most frequent cognitions were: descriptions of the lockdown effects on detention, descriptions of the prison during the COVID-19 emergency, the commitment of people in prison, evaluations of the virus and the riots, requests to the institutions (e.g. the Italian Minister of Justice).

A complete list of topics and subtopics for both cognitions and emotional connotations are provided, respectively, in Tables 1 and 2.

### Cognitions

In the 27 posts, participants wrote about cognitions 79 times. Cognitions were coded into four subtopics: descriptions, evaluations, commitment and requests to institutions. Each is explained in detail below.

### Descriptions

Descriptions were the most frequent (42%, 33/79) topic among cognitions. Participants provided detailed descriptions especially of the *lockdown effects on detention* (14/33) and of the *prison during the COVID-19 emergency* (10/33). They also provided information about the *COVID-19 situation in general* (7/33) and made a *comparison between inside and outside the prison* (2/33). Descriptions of *the lockdown effects on detention*, *the prison during COVID-19 emergency, COVID-19 pandemic situation in general* and *comparison between inside and outside the prison* represent subtopics of descriptions. Descriptions of *the lockdown effects on detention* (14/33) were the most frequent as people wrote predominantly about suspended visits with their relatives (7/14). The second most frequent was *cell closure for most of the day* (5/14) except for exercise time and the few minutes spent on the phone with loved ones:

In this period when visits are not allowed, we're locked in a cell all day except for exercise time. (ID 8, 29 y/o)

I stay in my cell 24 hours a day, except for those minutes dedicated to the phone calls with my loved ones. (ID 6, 56 y/o)

Finally, two participants wrote about the *suspension of group therapy* (2/14), essential for those people who follow a therapeutic program for the treatment of substance addiction on a daily basis:

This epidemic suspends the opportunity we had to treat ourselves (ID 4, 37 y/o)

Concerning descriptions of the *prison during the COVID-19 pandemic*, participants wrote about their daily life routine (6/10) and the difficulty in the application of restrictive measures within the prison, especially due to structural problems (4/10):

As usual, I wake up at 8:00 have a coffee and a cigarette, then the cell remains all for me since my cellmate gets exercise time. I look out of the window, at the street close to the prison: I have never seen it so deserted. (ID 6, 56 y/o),

Here, in prison, it's not easy to maintain social distances, to have the right masks and to sanitize everything. (ID 11, 31 y/o)

### Commitment

Despite the reduction of activities carried out in the ward – especially those in groups – due to the restrictions applied, participants demonstrated a good degree of commitment to dealing with the emergency. Commitment during the emergency represented 27% (21/79) of cognitions and 55% (15/27) narratives included sentences of commitment and encouragement. Shown below are two of the most significant:

Just do not give it too much weight and continue living your life. You must confront it and never give up in the face of difficulties. As my father has always taught me, be strong and brave, react and move forward. (ID 3, 36 y/o)

We try to keep in tune with our cellmates and manage our days by talking, writing, reading. I have been a self-appointed chef, creating new and challenging dishes. (ID 9, 42 y/o)

### Evaluations

One of the more frequent topics (25%, 20/79) categorized under cognitions was “evaluations”. Prisoners gave subjective evaluations mostly about *the virus* (9/20), *the riots* that happened in their section (7/20), *the circulation of information relating to the virus* (3/20) and the *COVID-19 situation worldwide* (1/20). *The Virus* was especially evaluated as “unexpected”. Other adjectives used are “unknown” and “temporary”. It was not only defined as an “inhuman killer” or an “evil which causes victims” but also “created by humans”. Participants also wrote about *the riots*, giving their evaluation on the causes and noting their unpredictability:

Everything was going well until the day of the riots, completely unexpected, which aggravated our living conditions. (ID 15, 25 y/o)

I do not think it was a form of protest against the restrictions due to the coronavirus, but a way to destroy something beautiful and positive created and wanted by all members of the team (ID 7, 53 y/o)

Other participants evaluated the riots as a “deplorable” act done by “a few people who, full of hatred for the institutions, tried to sink us” (ID 12, 35 y/o). In regard to *the circulation of information relating to the virus*, detainees wrote that information was insufficient and commented on how this affected people’s opinion and cognitions:

We're uninformed in here, and everyone has his say, often exaggerating. (ID 4, 37 y/o)

I was trying to understand what was going on without understanding much. It was the first time I heard the words ‘quarantine’, ‘asymptomatic’ and so on. Terms that were thundering in my head every day. (ID 18, 33 y/o)

## Requests to institutions

The least frequent topic (6%, 5/79) categorized under cognitions was represented by explicit “requests to the institutions”. The term “institutions” not only includes institutional bodies in general but also the Ministry of Justice and the current Italian Minister of Justice. Such requests included asking the institutions to consider granting people in prison extraordinary measures in terms of finalizing their sentences given the emergency:

I would expect the Justice Department to carefully consider the possibility of a pardon or, indeed, of house arrest before the virus prevails even in prison or other riots break out. (ID 14, 46 y/o)

We appeal to the penal institution to understand the suffering of being shut away almost 24 hours a day in cells of 3 by 4 meters, distressed by what is happening to our loved ones. (ID 6, 56 y/o)

## Emotional connotations

Participants wrote about emotional connotations 94 times in their 27 blog posts, making it more frequent than “cognitions” (*n* = 79). Emotional connotations were divided into positive and negative. Despite a good percentage of positive emotional connotations (31%), most of the emotional connotations expressed were negative (69%). All subtopics associated with positive and negative emotional connotations are presented below.

### Positive

Positive emotional connotations represent 31% (29/94) of posts characterized by emotions related to the experience of the COVID-19 pandemic in prison. Positivity was demonstrated mainly through messages of *hope* (66%, 19/29), and thanking the *prison workers for their support* (24%, 7/29). A smaller part (10%) covered: *trust* in our rulers (i.e. the government), *empathy* with those people who have suffered the loss of loved ones and *faith.* Regarding *hope*, people in prison especially wrote about a generalized hope (26%, 5/19) or about the hope to return to their previous life (5/19) before the advent of the pandemic. Hope has been directed also *towards the institutions*. Participants expressed the hope to be taken into consideration during the emergency (3/19):

The hope is that the institutions will do something more concrete for all of us. (ID 8, 29 y/o)

Furthermore, hope was addressed for the *end of infection*, the *improvement of medical treatment* needed to face the virus, for the *future*, for *a better situation* and *freedom*. Other than hope, the most frequent subtopic of “positive emotional connotations” was gratitude towards *prison workers*’ *support* (24%, 7/29). At that time, detainees could no longer do anything during the day and had no contact with anyone outside, except for a phone call once a week. Therefore, some of the participants turned directly to the prison workers in their section, thanking them for their support during the most difficult times of the pandemic:

Here, we are very lucky. Every day our counsellors show up on time and we continue treatment, even if it is reduced. We always see them smiling at us, trying to minimize the problem, giving us hope and bringing us news. They are always present to listen to our problems; the efforts they make for us must be recognized. (ID 6, 56 y/o)

### Negative

As anticipated, negative emotional connotations (69%, 65/94) were expressed more than positive ones. Due to the plurality of subtopics addressed and linked to the negative emotional connotations, only the most frequent (more than five occurrences) are presented below, supported by some of the citations. A complete list of subtopics is presented in Table 2.

The most frequent subtopics of “negative” emotional connotations are: *missing* (17%, 11/65); *worry* (15%, 10/65); *psychological pain* (14%, 9/65) and *fear* (10%, 7/65). *Missing* was expressed towards several objects, listed in order of frequency: *things taken for granted* and *contact, freedom, prison activities and hope.* In respect of “things taken for granted”, participants referred to things from their previous prison routine, especially exercise time:

I'm eagerly awaiting exercise time, which has now assumed a different importance than in the recent past, when we took it for granted. (ID 2, 53 y/o)

Missing of contact is also recurrent:

Having no contact with our family members is heart-breaking. (ID 6, 56 y/o)

Less frequent are *missing of freedom* (probably expressed regardless of the situation caused by the pandemic) and *missing prison activities*. Finally, in contrast to the results presented so far, one participant referred to the *missing of hope:*

We feel like we've been sucked into a hopeless vortex. (ID 10, 59 y/o)

Participants’ *worry* was predominantly focused on their relatives, partners and friends. The focus of worry shifting to relatives can be explained by the loss of contact with the outside world. The application of restrictive measures had caused a drastic reduction in contact with loved ones, including by telephone:

The only big worry I have is about my family, especially my little ones. (ID 4, 37 y/o)

Worry was also aimed at the victims of the pandemic and the infected:

“[…] to this was added the worry of the Coronavirus, with all its dead people and the many who have been infected. We suffer a great deal especially for our families that, to avoid contagion, we can no longer see. (ID 15, 25 y/o)

With a significantly lower percentage than *worry* (15%), participants described their suffering (14%). Specifically, they wrote about their *psychological pain* as a result of others becoming victims of the pandemic, but they also referred to a more generalized psychological pain:

I collapse into endless crying of a never before experienced pain. I can feel the pain and suffering that one feels when a son, a mother, a father, a brother or a man fall prey to this ravenous killer which makes no distinction of class as if evil had put us in a mortal grip. (ID 10, 59 y/o)

Other sufferings are in addition to what we already had. (ID 14, 46 y/o)

Finally, another frequent subtopic of negative emotional connotations is *fear (*10%). The CA indicated that the most common fear in this group of prisoners is the *fear of being infected*. Then, *fear of the infection outside*, fear *of death* and *of not getting over the current situation*:

I'm scared I'm gonna get the coronavirus. We feel like we're no longer serving time, but risking our lives every day. (ID 13, 26 y/o)

The remaining 43% of the negative emotional connotations’ content is represented by the following subtopics: malaise related to detention during COVID-19 (6%), impotence (4%), tension (4%), mistrust of other prisoners (3%), indignation towards those who do not respect the restrictive measures (3%), anxiety caused by the pandemic (3%), confusion and difficulty in expressing one’s emotions in words (3%), uncertainty about the future (3%), feelings of abandonment (3%), discouragement and regret for the consequences of the prison riots (2%), anger at the current situation (2%), struggle to stay rational (2%), fragility (2%), mood instability (2%) and alarmism (2%).

## Discussion

In the current study, the experience of 17 prisoners living in a Lombardy prison during the COVID-19 pandemic was explored using the qualitative CA of 27 narratives posted on “L’Oblò”, the institutional blog of “La Nave” ward in San Vittore prison (Milan, Italy).

Previous research highlights how narratives can help people cope with a stressful experience (Saita and Acquati, 2020) and can help researchers understand how people live life experiences (McAdams, 2012). Narratives have been used in the forensic field and are considered effective in prison populations because they allow prisoners access to the world, and therefore, provide them with a sense of stability and unity (Saita, 2018). In this case, the CA of narratives could be used to help prison administrations and policymakers better understand situations of crisis and develop more effective solutions and programs that address the issues and needs of prisoners. The current research led to the identification of two dominant categories of themes addressed in participants’ posts: *cognitions* and *emotional connotations* linked to the COVID-19 experience in prison.

Contents expressing the individual percepts, thoughts, ideas, opinions and explanations, related to the COVID-19 pandemic were assigned to “cognitions”. Cognitions have been defined by Neisser (1967) as all processes by which sensory input is transformed, reduced, elaborated, stored, recovered and used. Cognitions involve perceiving, conceiving, remembering, reasoning, judging, imagining, problem-solving (APA, 2020). According to Jackson and Crosson (2006) by “emotional connotations”, we mean the emotional property of an object, action or event. It is not a direct reference to an emotion such as happiness, sadness, anger or fear. It is implied knowledge that an object, action or event is likely to evoke a specific emotion. In our case, the event that produced emotions is the COVID-19 pandemic.

Cognitions were divided into four topics: descriptions, evaluations, commitment and requests to institutions, whilst emotional connotations were divided into positive and negative. The 27 blog posts written by the participants are mainly characterized by negative emotional connotations related to the experience of the COVID-19 pandemic in prison. Nevertheless, there are aspects in both cognitions and emotional connotations that are worth discussing in more detail.

Although, as expected, most of the participants’ cognitions were concentrated on the description and evaluation of the pandemic in prison, they also demonstrated a certain degree of commitment to dealing with the situation. In total, 15 out of 27 posts displayed how those in prison tried to be hopeful and positive, despite the distressing situation they were living in as one of the most vulnerable population groups. As pointed out by the WHO (2020), people in prisons typically have a greater underlying burden of disease and detrimental health conditions than the general population. They frequently face greater exposure to risks such as smoking, poor hygiene and weak immune defence due to stress, poor nutrition or prevalence of coexisting diseases, such as bloodborne viruses, tuberculosis and drug use disorders (Coninx *et al.*, 1995; Macalino *et al.*, 2004).

Despite all these difficulties, the participant’s positive attitudes could be related to the availability of information about the virus that they received. Although two participants evaluated information as “insufficient”, is it known that since the beginning of February, the Penitentiary Health Systems of Milan launched an information campaign in local prisons concerning the ongoing health emergency. In San Vittore, infectious diseases specialists with the help of nurses and prison officers have been meeting prisoners, ward by ward, offering explanations and carrying out awareness-raising activities, which have often been consolidated with the involvement of certain prisoners. In addition, leaflets have been distributed and placed in different languages throughout all wings. Workers of “La Nave” ward have confirmed that an awareness-raising campaign on flu and anti-pneumococcal vaccination is currently underway. It is well known that receiving information about COVID-19 could prevent psychological distress or anxiety (WHO, 2020), whilst a paucity of information can produce high psychological distress for prisoners (Okoro *et al.*, 2020). For “cognitions” an interesting issue was the way detainees evaluated and represented the virus to themselves. It is easy to imagine how something unknown like COVID-19 could be represented in a distorted or exaggerated way (Büchi *et al.*, 1998; Tosoli *et al.*, 2011). Participants defined it as “an ‘inhuman killer”, “created by humans” and an “evil which causes victims”, whilst some attributed confused and unrealistic qualities to the virus. Often, a negative image of the virus was linked to a more anxious outlook.

Other important aspects to be discussed can be found within the category “emotional connotations”. Even though negative emotional connotations were more frequent, among the positive ones there were messages of hope and gratitude for the support given by the prison workers. Our sample is entirely constituted by people struggling with substance use disorder and who follow a special treatment program, as members of “La Nave” ward. Unlike other wards of San Vittore, their treatment activities had not been completely suspended. As pointed out by the participants, group activities were interrupted due to social distancing, but individual sessions continued to provide psychological support. This is in line with recommendations not to interrupt any psychological support to the prison population even during a world health emergency (Liebrenz *et al.*, 2020).

Despite these efforts, several negative emotional connotations were felt by participants during the lockdown. The most frequent were missing, anxiety, psychological pain and fear. Missing was expressed by participants not only towards their family members due to the suspension of the visits but also towards their previous habits in the prison. Missing their daily routine was often felt, accompanied by psychological pain and disorientation to underline the difficulty of adaptation.

As highlighted by literature, isolation and a reduction in social interactions are dangerous for a person’s mental health and confinement in a cell for many hours a day with very few social contacts (as during the pandemic) can cause strong emotional disturbances (Haney, 2018). Psychological reactions due to isolation are, for example, stress-related reactions, high levels of anxiety, confusion, loss of emotional control and mood swings (Smith, 2006; Arrigo and Bullock, 2008).

Similar consequences have been found in the posts written by the individuals of our sample. Texts report emotions such as anxiety, confusion, difficulty in expressing one’s emotions in words, mood instability, struggling to stay rational, fear and anger. These emotions appear plausible considering, for example, that social distancing to reduce the risk of contagion is not feasible in prisons because people are confined to small living spaces and institutions are often over capacity (Wurcel *et al.*, 2020; Dumont *et al.*, 2012; Marushak *et al.*, 2009).

It is well known that the need and importance of psychological interventions in prisons are beneficial in many ways. People who do not receive psychological treatment have a major risk of recidivism after release (Mears and Cochran, 2012; Baillargeon *et al.*, 2009); psychological health improves in those who have followed group therapy (Khodayarifard *et al.*, 2010); psychopathological symptoms have been shown to decrease after prisoners have received psychological support (Shelton *et al.*, 2009).

Despite the gratitude expressed for the attention and support delivered by prison workers, participants pointed out discontent concerning suspended activities (especially the group ones). The interruption of treatment activities and visits from families could have a negative psychological impact on the precarious emotional balance of this fragile population already deprived of freedom. Consequences could be devastating as demonstrated in Italian prisons (including San Vittore), where violent riots occurred following the tightening of contagion risk containment measures.

It was not the aim of the current study to analyse the reasons for the riots; however, several negative connotations in the posts led us to presume that the changes enforced by the pandemic generated negative consequences. Even if adjustments are necessary, often they are applied without full consideration of the habits that characterize the context (Saita, 2018). Semenza and Grosholz (2019) observed that those in prison dealing with concurrent mental and physical health troubles have a higher probability to engage in misconduct than healthy individuals. We can suppose that the COVID-19 related negative emotional connotations, expressed by the people living in San Vittore, was likely to lead to violence.

Given the deprived conditions and its specific clinical features, the prison population are perhaps prone to develop psychological distress because of an event that we can define as catastrophic. However, the analysis of the participants’ narratives, written during the COVID-19 pandemic, confirmed:

- how important information on what is happening is and the overall importance of communication;
- that continuity of psychological support provided by the professionals (although compromised by anti-contagion standards) should be considered as an important protective factor that allowed detainees to voice their feelings and professionals to understand and treat the participants’ emotional states.

## Limitations

The current study presents several limitations. One is represented by the small number of participants and posts that form the basis of the analysis. Moreover, we included posts collected from only one blog. The rationale behind the use of “L’Oblò” is that it is the only blog updated from both prisoners and prison workers of a prison within the Lombardy region (i.e. the Italian region most affected by the COVID-19 pandemic). Beyond that, the experience narrated by the participants is unique because of the particularity of the ward where they are hosted. At “La Nave”, special attention is given to the psychological treatment of people with substance use disorder.

Even though every one of the wards is free to participate in writing on the blog, without incurring any consequences, the sample comprises a selected number of individuals. It is limited to those people who choose to participate in writing on the blog and who can write well enough in Italian. Moreover, the analyses were conducted on posts published during a specific period, corresponding to the initial phases of the pandemic in Italy. Over time, things might have changed because of progressive adaptation to the situation.

## Implications

Despite the limitations listed above, this study made it possible to raise awareness about a low-cost and easy-to-use tool/means (i.e. the blog) that can help people in prisons to record their experiences and to make their voices heard. At the same time, the study highlights the need to think about and provide this population with more opportunities for self-expression, which will help to shorten the distance between inside and outside the prison.

In this case, better understanding the experiences of people living in an Italian prison during the COVID-19 pandemic allows us to consider information that can lead to improvements for future experiences in the detention context. Indeed, there is a need to coordinate both the health and the justice services for supporting people in prison during a pandemic. Moreover, prison health-care staff must monitor the needs of people in prisons and try to respond practically or communicate better to prevent riots and violence. As mental health services in the community played and still play an essential role in helping their patients during this pandemic, it is equally important to enhance mental care for the most vulnerable. In fact, despite the negative feelings that the experience of the pandemic has caused, being located in a prison ward with a higher level of attention (because of the drug addiction treatment program) allowed our participants to experience a positive attitude.

The psychological experience of positive elements such as hope can be a protective factor in emergencies (Demetriou *et al.*, 2020). Among our sample, hope seems to have been encouraged both by the psychological support that prison workers have continued to provide (despite the limitations) and the information and awareness campaign on the virus promoted by the Milan Prison Health System. Our results support a clear need for additional psychological support for people in prison, promoted at an individual level and, if necessary, with alternative provisions such as the telephone or computer when confronted with a reduction in staff.

Resources invested in this way could reduce the negative impact of a catastrophic event on the already impaired health of this fragile population. Underestimating the importance of these choices can expose judicial systems to serious consequences such as those that occurred in Italy.

Finally, this study provides useful information that contributes to the literature by discussing the psychological effects of a world health emergency such as the COVID-19 pandemic among those in prison. Through blog posts, it has been possible to analyse the subjective experience of a population often ignored or considered unworthy of being heard.

## Figures and Tables

**Table 1:** Topics and subtopics related to the category “cognitions”

Topics	Subtopics	Details
Descriptions	Lockdown effects on detention	- Visits with relatives suspended - Cell closure –Group therapies suspended
	Prison during the COVID-19 emergency	- Daily life routine - Difficulty in application of restrictive measures in prison
	The COVID-19 pandemic situation in general	
	Comparison inside/outside the prison	
Commitment		
Evaluations	Virus	- Unexpected - Unknown - Temporary - Inhuman killer - Created by humans - New –An evil which causes victims
	Riots	- Unpredictable - Causes - Negative opinions about the riots
	Circulation of information relating to the virus	- Insufficient information - Information influence
	The COVID-19 situation Worldwide	
Requests to institutions		

**Table 2:** Topics and subtopics related to the category “emotional connotations”

Topics	Subtopics	Details
Positive	Hope	- Generalized - Return to the previous life - Towards the institutions - End of infection - Improvement of medical treatment - Future - Freedom - Better situation
	Support of prison workers	
	Trust	
	Empathy	
	Faith	
Negative	Missing	- Things taken for granted - Contact - Freedom - Prison activities - Hope
	Worry	- Relatives/partners/friends - Victims and infected
	Psychological pain	- COVID-19 victims - Generalized
	Fear	- Being infected - Infection outside - Death - To not get over the situation
	Malaise related to detention during COVID-19	
	Impotence	
	Tension	
	Mistrust	Of other prisoners
	Indignations– towards those who do not respect restrictive measures	
	Anxiety	
	Confusion and alexithymia	
	Feeling of abandonment	– By institutions
	Discouragement and regret	– For the consequences of the riots
	Anger	
	Struggle to stay rational	
	Fragility	
	Mood instability	
	Alarmism	

## Notes

1.
www.gnewsonline.it/carceri-bonafede-12-detenuti-morti-40-agenti-feriti-e-strutture-danneggiate/

2.
www.gnewsonline.it/carceri-22-istituti-interessati-da-rivolte-e-disordini/

3.
www.medicisenzafrontiere.it/news-e-storie/news/coronavirus-aggiornamento-sullepidemia/#italia

4.
http://oblodelanave.blogspot.com
